# Proteomics provides insights into the inhibition of Chinese hamster V79 cell proliferation in the deep underground environment

**DOI:** 10.1038/s41598-020-71154-z

**Published:** 2020-09-10

**Authors:** Jifeng Liu, Tengfei Ma, Mingzhong Gao, Yilin Liu, Jun Liu, Shichao Wang, Yike Xie, Ling Wang, Juan Cheng, Shixi Liu, Jian Zou, Jiang Wu, Weimin Li, Heping Xie

**Affiliations:** 1 Department of Otolaryngology Head and Neck Surgery, West China Hospital, Sichuan University, No. 37 Guoxuexiang, Chengdu, China; 2 Deep Underground Space Medical Center, West China Hospital, Sichuan University, Chengdu, China; 3 College of Water Resources & Hydropower, Sichuan University, Chengdu, China; 4 Department of Ophthalmology, West China Hospital, Sichuan University, Chengdu, China; 5 Institute of Deep Earth Science and Green Energy, Shenzhen University, Shenzhen, China

**Keywords:** Biophysics, Cell biology, Environmental sciences

## Abstract

As resources in the shallow depths of the earth exhausted, people will spend extended periods of time in the deep underground space. However, little is known about the deep underground environment affecting the health of organisms. Hence, we established both deep underground laboratory (DUGL) and above ground laboratory (AGL) to investigate the effect of environmental factors on organisms. Six environmental parameters were monitored in the DUGL and AGL. Growth curves were recorded and tandem mass tag (TMT) proteomics analysis were performed to explore the proliferative ability and differentially abundant proteins (DAPs) in V79 cells (a cell line widely used in biological study in DUGLs) cultured in the DUGL and AGL. Parallel Reaction Monitoring was conducted to verify the TMT results. γ ray dose rate showed the most detectable difference between the two laboratories, whereby γ ray dose rate was significantly lower in the DUGL compared to the AGL. V79 cell proliferation was slower in the DUGL. Quantitative proteomics detected 980 DAPs (absolute fold change ≥ 1.2, *p* < 0.05) between V79 cells cultured in the DUGL and AGL. Of these, 576 proteins were up-regulated and 404 proteins were down-regulated in V79 cells cultured in the DUGL. KEGG pathway analysis revealed that seven pathways (e.g. ribosome, RNA transport and oxidative phosphorylation) were significantly enriched. These data suggest that proliferation of V79 cells was inhibited in the DUGL, likely because cells were exposed to reduced background radiation. The apparent changes in the proteome profile may have induced cellular changes that delayed proliferation but enhanced survival, rendering V79 cells adaptable to the changing environment.

## Introduction

As resources in the shallow depths of the earth become exhausted, people will spend extended periods of time living and/or working in the deep underground space, reaching historical depths^1,2^. Currently, deep mining is common, with exploitation of metal resources continuing to more than 4000 m deep in a gold mine in South Africa^2^. However, little is known about the environmental factors that might affect the health of humans or other organisms that live or work in the underground space, especially deep underground^1^.

Several researchers have investigated the effects of low background radiation on living organisms maintained in deep underground laboratories (DUGLs)^3,4^. Eugster et al. observed that the cyanobacterium *Mastigocladus laminosus* cultured in the Simplon tunnel (2000 m of rock cover) died after a few weeks^5^. Other researchers found less dramatic effects, reporting reduced growth rates in paramecium, bacteria, human lymphoblastoid TK6 cells and Chinese hamster V79 cells cultured in the deep underground environment and/or when shielded from cosmic radiation^6–10^. Some studies showed no apparent difference in growth rates in TK6 cells and V79 cells cultured in the Gran Sasso National Laboratory (LNGS) compared to a control environment^9,10^. The reasons for these contrasting results remain to be elucidated^3^. Fortunately, research into the biological effects induced by the deep underground environment has attracted the attention of DUGLs worldwide, which were traditionally used for rare event experiments due to the virtual absence of cosmic rays^1^. Consequently, the LNGS, the Waste Isolation Pilot Plant (WIPP), the Sudbury Neutrino Observatory Laboratory (SNOLAB), the ANDES underground laboratory (ANDES) and the Deep Underground Science and Engineering Laboratory (DUSEL) have conducted or are planning to conduct biological experiments.

**Figure 1:**
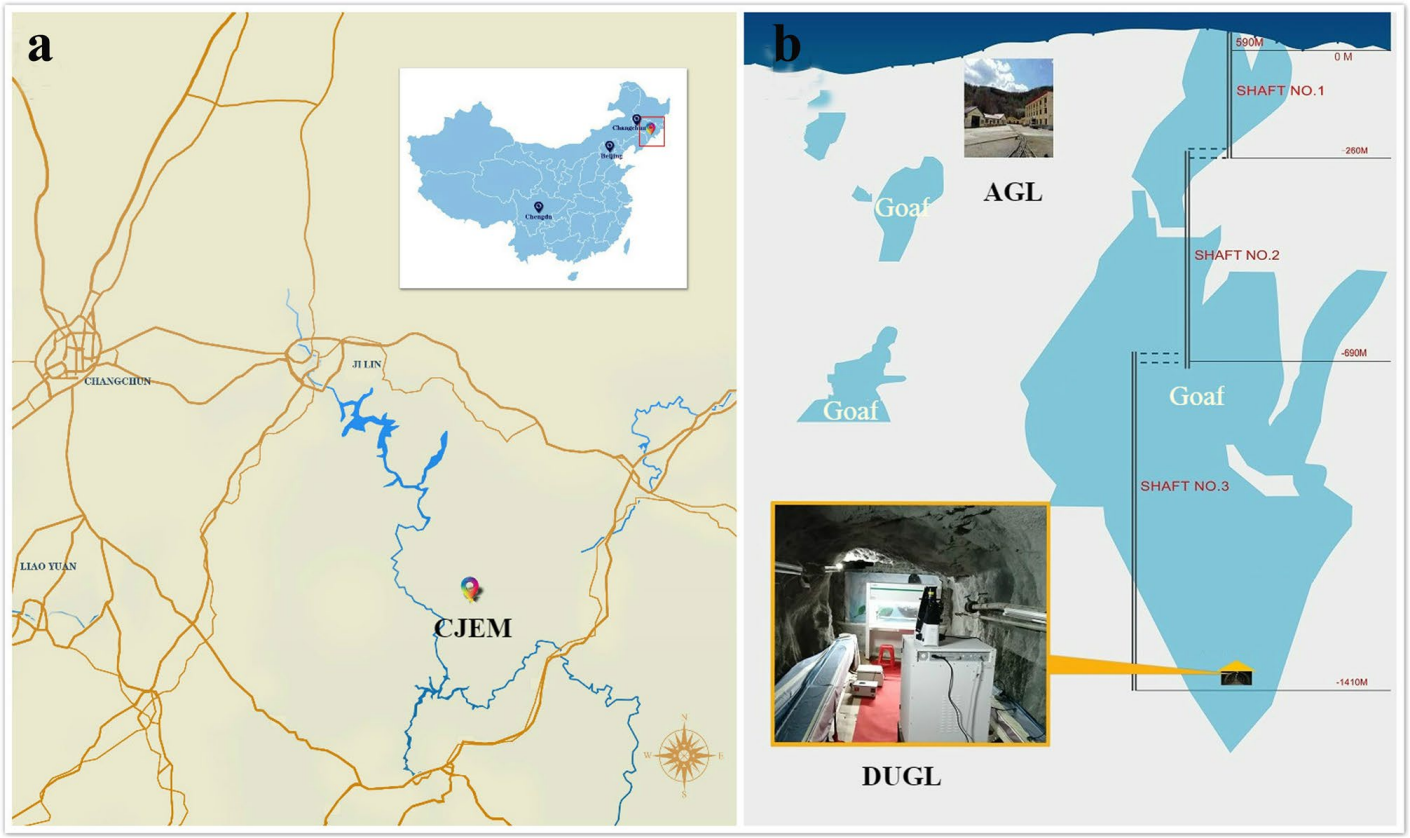
The location of the DUGL and the AGL at the CJEM. The location of the CJEM in China (**a**), and the location of the DUGL and the AGL in the CJEM (**b**). AGL, above-ground laboratory; CJEM, Erdaogou Mine, Jiapigou Minerals Limited Corporation of China National Gold Group Corporation; DUGL, deep-underground laboratory.(Adobe Photoshop CS3 was used creating (**a**), the URL is https://download.zol.com.cn/detail/35/347361.shtml).

In China, exploiting the deep underground space and resources has become a national priority^11^. Heping Xie advocated the need to harness beneficial elements and avoid factors that are potentially harmful to humans and other organisms in the deep underground environment^11^. A new discipline, deep underground medicine, has been conceptualized as a strategy to determine the effects and mechanism of action of factors in the deep underground space that may influence humans’ physiological and psychological health, and to implement appropriate countermeasures^1^. Under the guidance of Heping Xie, a deep underground medical laboratory has been established in Erdaogou Mine, Jiapigou Minerals Limited Corporation of China National Gold Group Corporation (CJEM) in Northeast China (Fig. 1). An above ground laboratory (AGL) in an office building near the entrance of the CJEM is being used for control experiments.

To observe the biological effect of low background radiation in a DUGL, a series of studies on V79 cells was conducted in the LNGS^9,10,12,13^. To test the feasibility of the DUGL at the CJEM, similar experiments using V79 cells were conducted in December 2017^1^. Initial findings showed that V79 cells could be successfully cultured in the DUGL. Here, we characterize the environment in the DUGL at the CJEM, and the effect of the environment in the DUGL on the growth and metabolism of cultured V79 cells. Our results indicated that proliferation of V79 cells was inhibited in the DUGL, likely because cells were exposed to reduced background radiation. There were apparent changes in the proteome profile that may have induced cellular changes that delayed proliferation but enhanced survival, rendering V79 cells adaptable to the changing environment. These data will provide new insight into the biological effects of the deep underground environment.

## Results

### Environmental parameters in the DUGL and AGL

**Figure 2:**
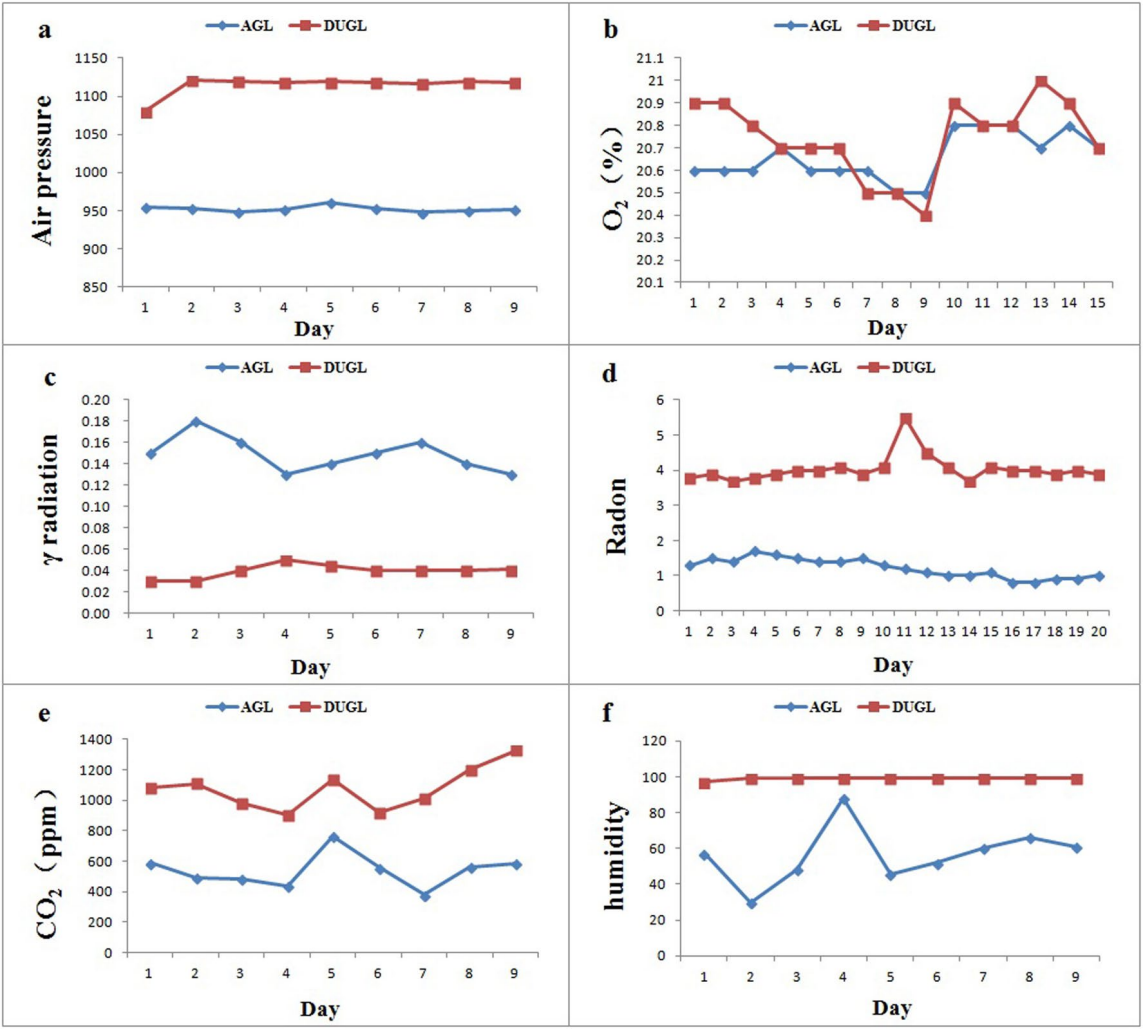
Variations in the environmental characteristics in the DUGL and AGL. *AGL* above-ground laboratory, *DUGL* deep underground laboratory.

As a newly established lab in deep underground environment, six environmental parameters of both the DUGL and control AGL were quantitatively characterized. Environmental parameters measured in the DUGL and AGL are summarized in Table 1 and Fig. 2. The following data are expressed as mean ± SD or median (interquartile range).O_2_ concentration in the DUGL[20.8% (20.7–20.9%)] and AGL [20.6% (20.6–20.8%) ] was not significantly different. Total γ ray dose rate was significantly lower in the DUGL[0.04μSv/h (0.035–0.045μSv/h) ] compared to the [0.15 μSv/h AGL(0.13–0.18μSv/h)] (*p* = 0.005). Relative humidity [DULG/AGL = 99% (99–99%)/57.2%(46.9–63.6%)] (*p* < 0.001), air pressure [DULG/AGL = 1,118.2 hPa (1,117.3–1,119.6 hPa)/ 951.9 hPa (949.65–953.9 hPa)] (*p* < 0.001), and concentration of CO_2_ [DULG /AGL = 951.9 ± 137.56 ppm/ 540.11 ± 110.39 ppm] and radon gas[DULG /AGL = 4.0 pCi/L (3.9–4.1 pCi/L)/1.25 pCi/L (1–1.47 pCi/L)] (*p* < 0.001) were significantly higher in the DUGL compared to the AGL. All parameters measured in the DUGL fluctuated over a small range.

**Table 1:** Environmental characteristics in the DUGL and AGL.

Environmental parameters	n*	AGL	DUGL	*p*
Air pressure (hPa)	9	951.9 (949.65–953.9)	1,118.2 (1,117.3–1,119.6)	< 0.001
O_2_ concentration (%)	15	20.6(20.6–20.8)	20.8 (20.7–20.9)	0.079
Total γ radiation dose rate (μSv/h)	9	0.15(0.13–0.18)	0.04(0.035–0.045)	0.005
Radon concentration (pCi/L)	20	1.25(1–1.47)	4.0(3.9–4.1,3.7–5.5)	< 0.001
CO_2_ concentration (ppm)	9	540.11 ± 110.39	951.9 ± 137.56	< 0.001
Relative humidity (%)	9	57.2 (46.9–63.6)	99 (99–99)	< 0.001

*DUGL* deep-underground laboratories, *AGL* above-ground laboratory.

Data are expressed as mean ± SD or median (interquartile range).

*Number of observations; each observation was made on a different day.

### Cell growth and morphology

**Figure 3:**
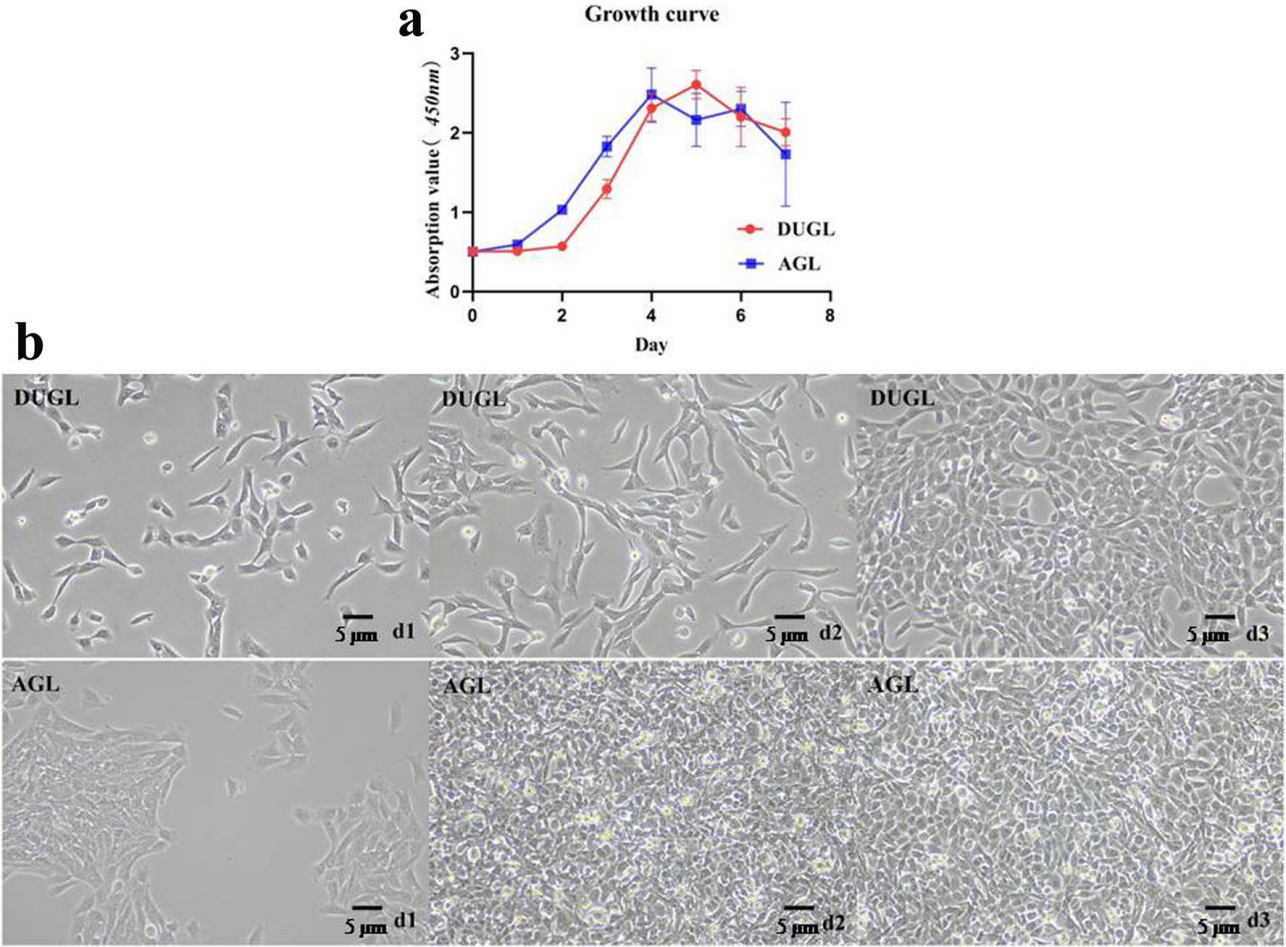
Growth curves of V79 cells cultured in the DUGL or AGL (**a**). V79 cells cultured for 3 days in the DUGL or AGL observed by light microscopy 10 × (**b**). *AGL* above-ground laboratory, *DUGL* deep underground laboratory.

To test the biological effect of the DUGL, the proliferation of V 79 cell was firstly analyzed by CCK-8. And which showed that V79 cell proliferation was slower in the DUGL compared to the AGL. After 2 days, V79 cell count had doubled in cultures grown in the AGL (OD value: AGL/DUGL = 1.03/0.503, *p* < 0.0001), but had only increased by 11.13% in cultures grown in the DUGL (OD value: AGL/DUGL = 0.572 /0.513). After 3 days, OD_*450*_ nm values obtained for V79 cells cultured in the AGL were approximately 1.5 times greater than those obtained for the DUGL (OD value: AGL/DUGL = 1.829/1.293), and the density of V79 cells cultured in the DUGL was obviously less than the AGL (Fig. 3). After 4 days, the growth curves of V79 cells cultured in the DUGL and AGL plateaued as the cells had reached maximal saturation density in the wells of the microtitre plates.

**Figure 4:**
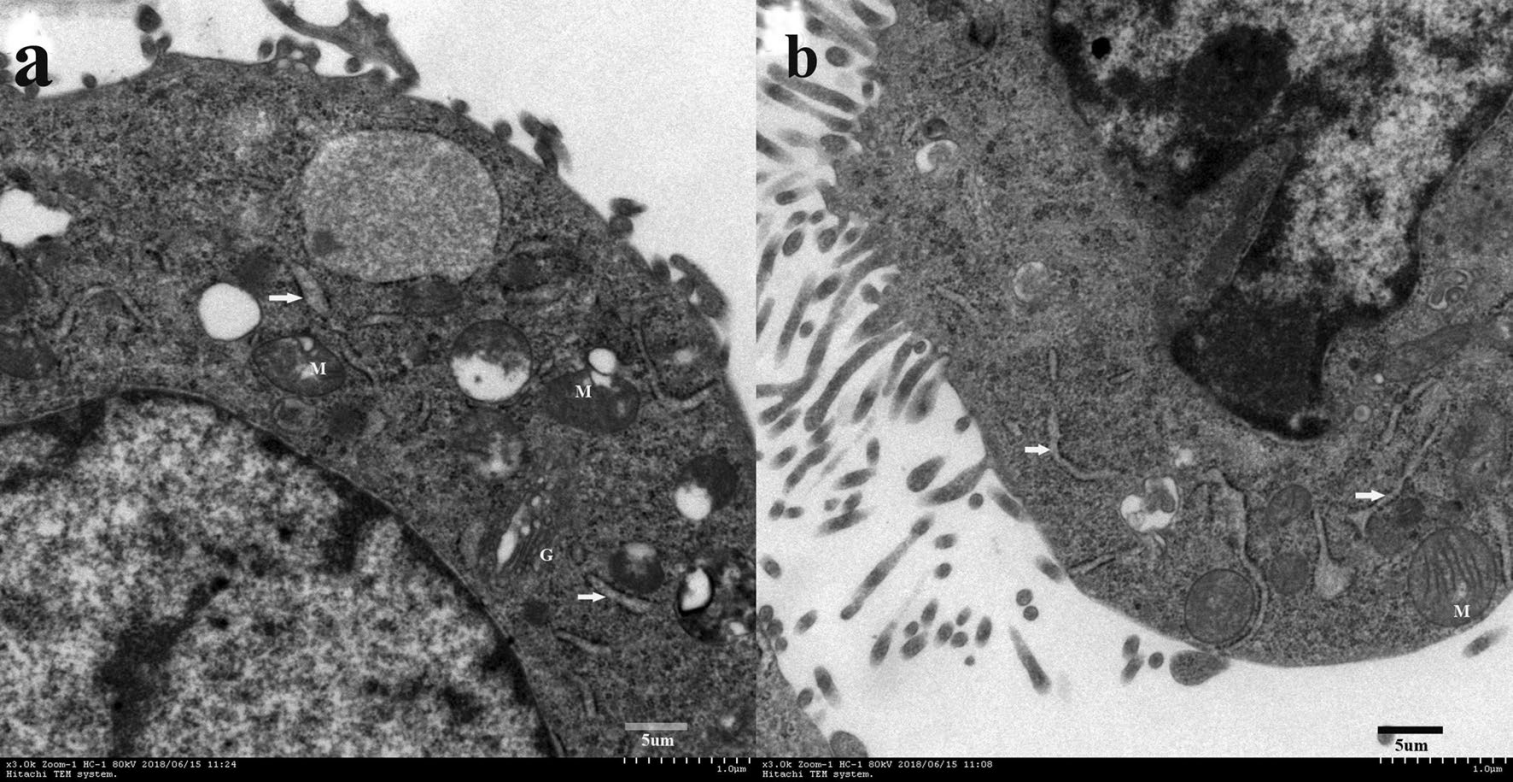
Transmission electron microscopy of V79 cells cultured in the DUGL (**a**) or AGL (**b**) 3,000 × White arrows: endoplasmic reticulum; *M* mitochondria, *G* Golgi body, *AGL* above-ground laboratory, *DUGL* deep underground laboratory.

In addition, TEM of V79 cells cultured in the DUGL showed that mitochondrial volume had increased compared to the AGL, mitochondria were largely devoid of cristae, and cells had a hypertrophic endoplasmic reticulum (ER) and obvious Golgi bodies (Fig. 4).

### Quantitative proteomic analyses

**Figure 5:**
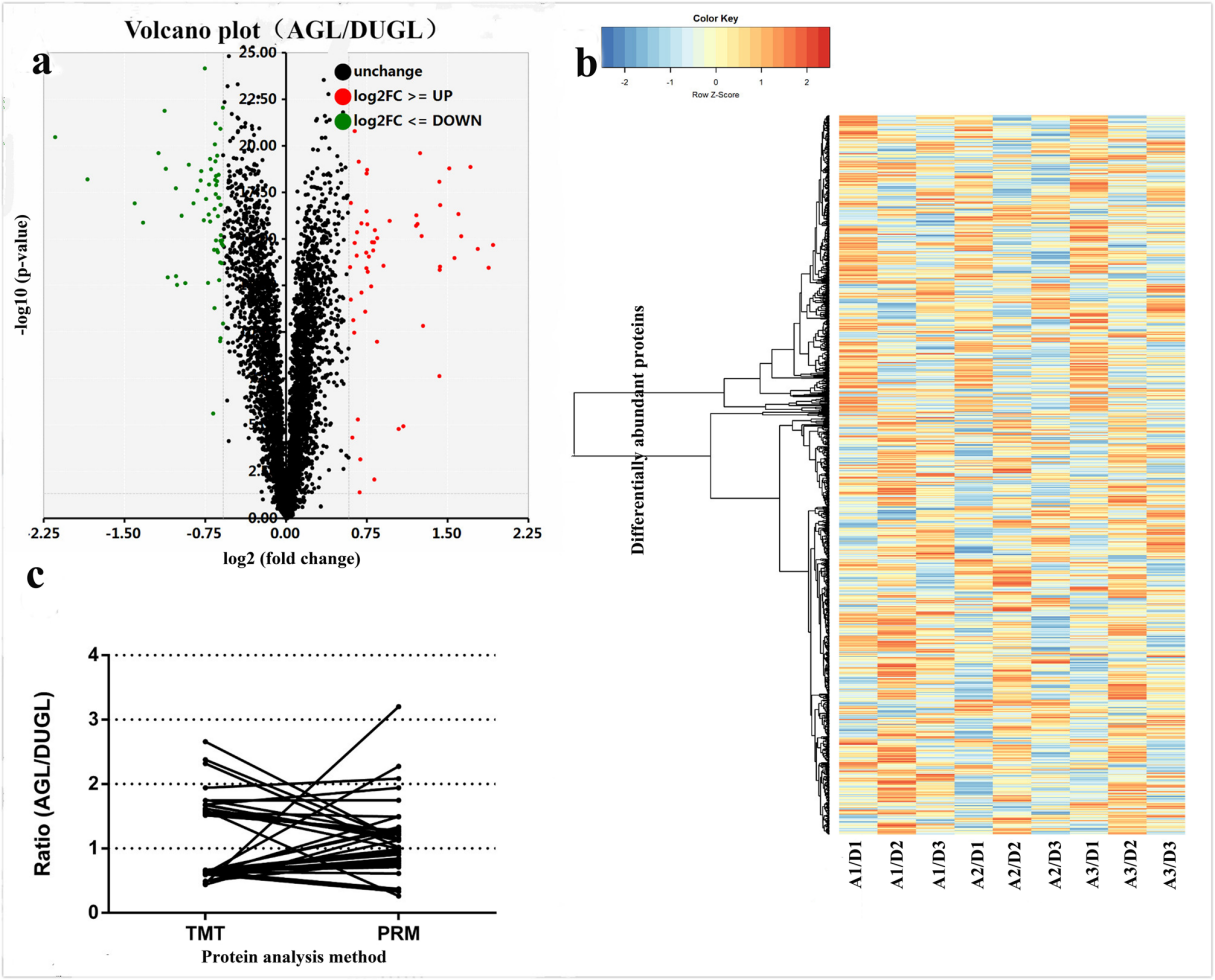
Volcano plot (red, up-regulated DAPs; black, unchanged DAPs; green, down-regulated DAPs [AGL/DUGL]) (**a**) and hierarchical cluster (white, unchanged DAPs; bright red, over-expression of DAPs [AGL/DUGL]) of DAPs in V79 cells cultured in the DUGL(**b**). Verification analysis shows selected DAPs verified by PRM (AGL/DUGL) (**c**). DAP, differentially abundant proteins; DUGL/D, deep underground laboratory; AGL/A, above ground laboratory; PRM, parallel reaction monitoring. The X-axis of (**c**), the DAPs of samples.

TMT proteomics analysis was performed to explore the molecular basis of V79 cells cultured in the DUGL. The quantitative proteomics detected 30,184 unique peptides (367,313 spectra, and 92,348 unique spectra) mapping to 4,622 unique proteins in V79 cells cultured in the DUGL and the AGL. A total of 980 differentially abundant proteins (DAPs), defined as proteins with a ≥ 1.2-fold change in relative abundance (*p* < 0.05) between V79 cells cultured in the DUGL and AGL, were identified. Of these, 576 proteins were up-regulated and 404 proteins were down-regulated in V79 cells cultured in the DUGL compared to the AGL (Fig. 5a,b and Supplementary Table S1). Protein names, abbreviations and accession numbers were obtained from the UniProtKB/Swiss-Prot database.

### Functional analysis of DAPs

The biological functions of the DAPs in V79 cells cultured in the DUGL were investigated using Gene Ontology (GO) term enrichment analysis and Kyoto Encyclopedia of Genes and Genomes (KEGG) pathway analysis. GO term enrichment analysis provided insight into the function of the DAPs, and KEGG pathway analysis was used to identify pathways for the DAPs^14^.

**Figure 6:**
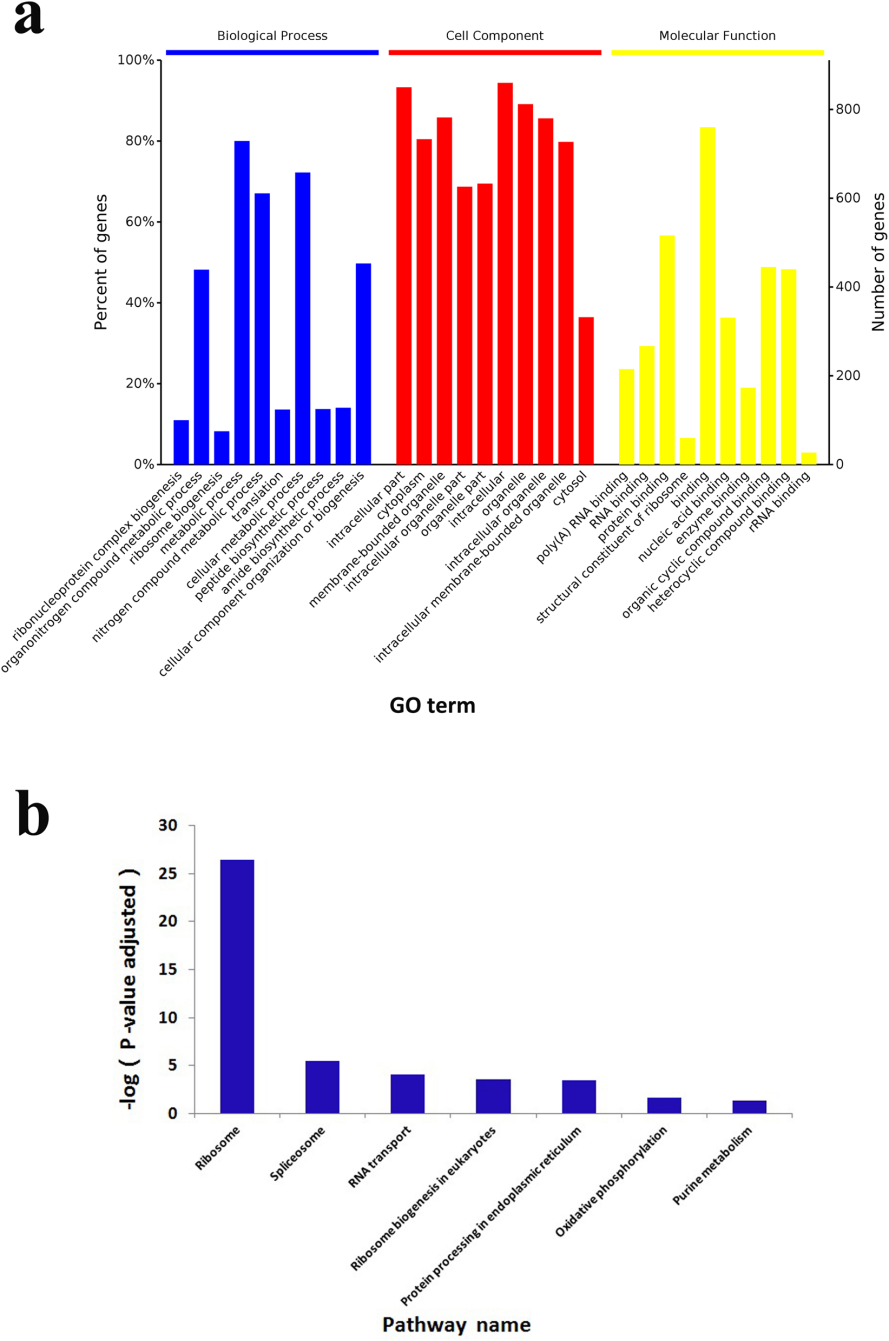
GO and KEGG enrichment analysis of differentially abundant proteins. (**a**) GO analysis result. (**b**) KEGG pathway analysis result.

GO term enrichment analysis of DAPs revealed the top five enriched GO terms were ribonucleoprotein complex biogenesis, organonitrogen compound metabolic process, ribosome biogenesis, metabolic process and nitrogen compound metabolic process in the biological process (BP) category; intracellular part, cytoplasm, membrane-bounded organelle, intracellular organelle part and intracellular organelle in the cellular components (CC) category; and poly(A) RNA binding, RNA binding, protein binding and structural constituent of ribosome and binding in the molecular functions (MF) category (Fig. 6a and Supplementary Table S2).

KEGG pathway analysis of the DAPs revealed that seven pathways were enriched. The significantly enriched pathways were ribosome (*p* < 0.0001), spliceosome (*p* = 0.0001), RNA transport (*p* = 0.0001), ribosome biogenesis in eukaryotes (*p* = 0.0003), protein processing in endoplasmic reticulum (*p* = 0.0004), oxidative phosphorylation (OXPPL) (*p* = 0.0242), and purine metabolism (*p* = 0.0416) (Fig. 6b and Table 2).

**Table 2:** KEGG pathway enrichment result.

Pathway	*p*-value (adjusted)	Up-regulated protein (n)	Name of up-regulated DAPs	Down-regulated protein (n)	Name of down-regulated DAPs
Ribosome	*p* < 0.0001	56	Rps18, Rpl18, Rplp0, Rpl8, Rplp2, Rps19, Rpl5, Rps13, Rpsa, Rpl13a, Rpl14, Rps4x, Rpl19, Rpl23, Rpl3, Rps24, Rpl15, Rps14, Rpl27, Rpl31, Rps16, Rps8, Rpl24, Rps3a, Rpl37, Rpl13, Rps15a, Rps23, Rps25, Rps27, Mrpl18, Rpl11, Rps17, Rpl28, Rps5, Rps9, Rpl26, Rpl27a, Rpl10, Rpl34, Rpl9, Rpl35, Rpl4, Rpl30, Rps11, Rps6, Rpl36a, Rpl6, Rps2, Rps21, Rps20, Rps28, Rpl18a, Rpl12, Rps26, Rsl24d1	0	
Spliceosome	0.0001	25	Srsf7, Rbm25, Ccdc12, Lsm8, Snrpg, Zmat2, U2surp, Prpf40a, Snrpd2, Rbm8a, Ppie, Cwc15, Dhx8, Snu13, Sf3a3, Slu7, Srsf5, Srsf6, Srsf3, U2af1, Ppih, Snrpf, Snrpd3, Sf3b4, Phf5a	3	U2af2, Snrnp40, Hnrnpm
RNA transport	0.0001	28	Eif4e, Nxt1, Snupn, Eif3c, Rnps1, Strap, Upf1, Eif4b, Eef1a1, Eif5b, Pnn, Phax, Eif3h, Rbm8a, Sap18, Nup37, Eif1, Eif5, Nup50, Eif2s1, Eif3j1, Eif3g, Srrm1, Rpp14, Smn1, Eif2s2, Nup153, Eif1a	1	Eif4a2
Ribosome biogenesis in eukaryotes	0.0003	18	Mphosph10, Rbm28, Nol6, Nxt1, Nvl, Wdr3, Wdr43, Rrp7a, Imp4, Utp14a, Snu13, Tcof1, Nob1, Gtpbp4, Nop58, Dkc1, Gnl3l, Gnl2	0	
Protein processing in endoplasmic reticulum	0.0004	4	Skp1, Dnaja2, Man1a2, Eif2s1	23	Wfs1, Stt3b, Pdia4, Canx, Tram1, Pdia3, Amfr, Erp29, Stt3a, Dnajc10, Syvn1, Hsph1, Capn1, Hspa5, Mogs, Casp12, Bcap31, Prkcsh, Pdia6, Ganab, Dnajb1, Dad1, Dnajc3
Oxidative phosphorylation	0.0242	8	Atp6v1g1, Uqcrh, Uqcrc1, Atp6v1b1, Atp5o, Atp5e, Atp5j, Atp5d	12	Mtnd4, Atp5l, Mtatp8, Ndufa3, Cox6b1, Mtco2, Uqcrfs1, Sdhb, Yjefn3, Ndufs4, Ndufb8, Atp6v0a2
Purine metabolism	0.0416	13	Hprt1, Rrm2, Nme2, Polr3g, Polr2h, Gmpr2, Polr1d, Polr2f., Twistnb, Pde6d, Pole3, Dck, Adcy6	10	Gucy2f., Xdh, Hddc3, Pgm1, Ampd2, Ampd1, Ampd3, Ak3, Polr2l, Pnp

### Verification of DAPs by parallel reaction monitoring

According to the fold change and abundance of those DAPs, 38 DAPs were selected to verify the result of TMT by parallel reaction monitoring (PRM). 76.23% (29/38) of these DAPs(e.g. G3H7U7, G3HHV4 and G3II46) identified by PRM were consistent with TMT proteomic analysis, suggesting that TMT proteomic analysis is reliable (Fig. 5c and Supplementary Table S3).

### Protein–protein interaction network construction and module analysis

**Figure 7:**
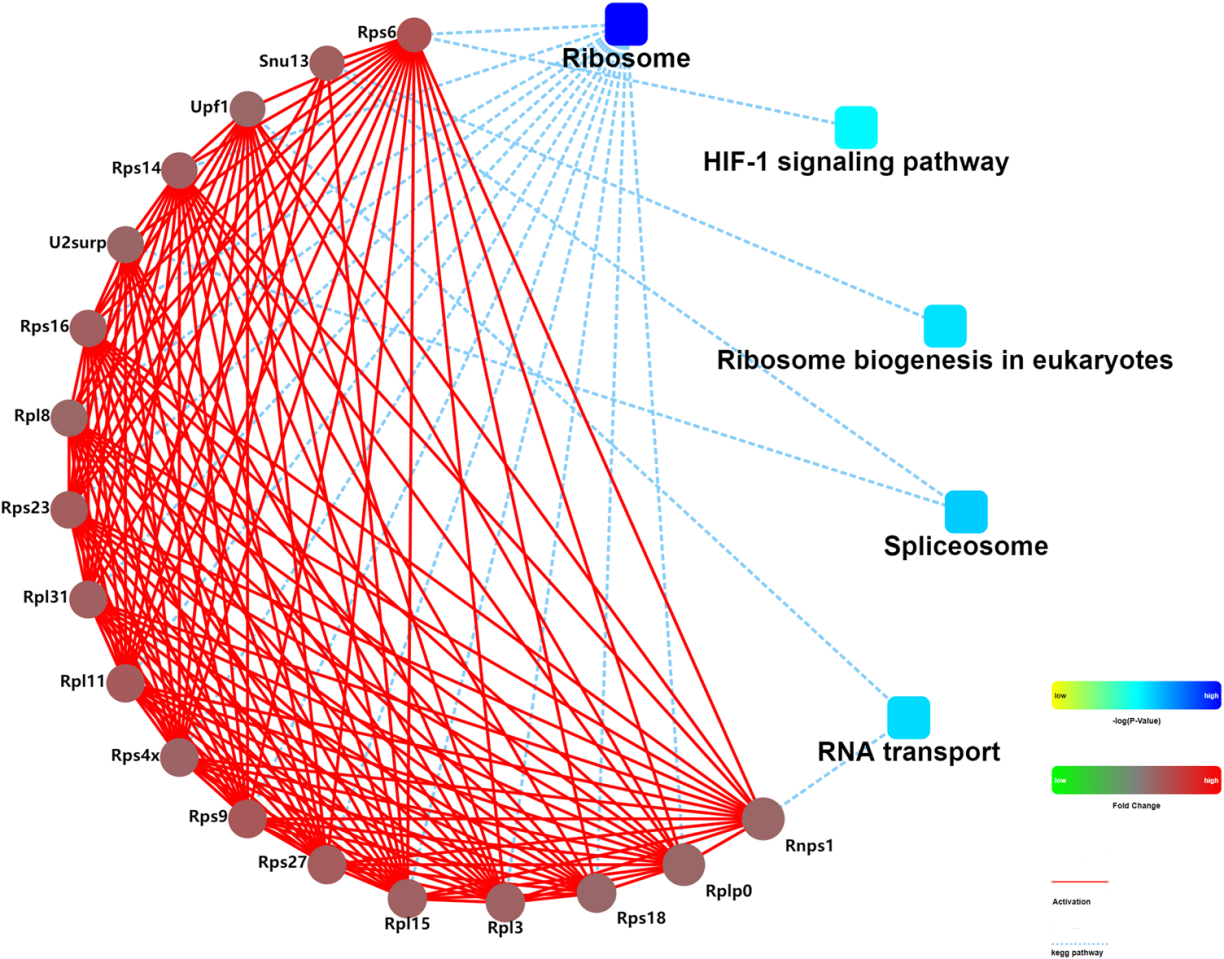
Protein–protein interaction(PPI) network of differentially abundant proteins in V79 cells. The analysis was based on the fold changes of differentially abundant proteins, PPIs and KEGG pathway. Circle nodes represent proteins. The rectangles represent KEGG pathways, colored with gradient colors from yellow (lower P-value) to blue (higher P-value). Proteins abundant changed are colored with red (up-regulation) and green (down-regulation). 400 was used as a default confidence cutoff. The red solid lines represent activation. The blue dashed lines indicate KEGG pathway.

To further ascertain functional interactions between DAPs, protein–protein interaction (PPI) networks were constructed using Cytoscape software (Fig. 7), with a confidence cutoff of 400. Findings showed that ribosome-related proteins were highly interrelated, playing key roles throughout the network.

## Discussion

This study quantitatively characterized environmental parameters in the DUGL at the CJEM and investigated the biological effects of these environmental parameters on V79 cells. This study provides the first research data to inform the new discipline of deep-underground medicine.

Six environmental parameters (radon gas, O_2,_ total γ ray dose rate, CO_2_, air pressure, relative humidity) with potential biological effects were monitored in the DUGL and the AGL. Relative humidity (99%), air pressure, and concentration of CO_2_ and radon gas were significantly higher in the DUGL compared to the AGL. The total γ radiation dose rate was significantly lower in the DUGL (0.03–0.05 μSv/h) compared to the AGL (0.13–0.18 μSv/h), even though radon gas is an important source of ionizing radiation. Compared to the LNGS, the concentration of radon gas was slightly higher in the DUGL at the CJEM, but total γ radiation dose rate and relative humidity were similar. Radon concentration in the DUGL was 1.5 pCi/L, which is less than normal background (1.7 nGy/h)^10^.

The present study confirmed previous reports that show reduced growth rates in cell lines within a short time (several days to two weeks) of being introduced to the deep underground^6–8,15^. Findings contrast with Satta et al. who found a significant increase in cell density at confluence in V79 cells grown in the LNGS compared to parallel populations cultured above ground^9,16^. These disparate results may be explained by dissimilar methodology. In the present study, cell proliferation was measured daily during the 7 days after V79 cells had been introduced to the DUGL, while Satta et al. observed their cells when they had been maintained in exponential growth in the LNGS for 9 months^9,16^. Short term stress responses in cells undergoing an acute environmental change differ from the adaptive response seen in cells exposed to chronic stress^17^. Cells cultured in the deep underground for many months may adapt to their environment and show no difference in proliferation rates compared to cells grown above ground^9,16^.

We speculate that reduced background radiation inhibited V79 cell proliferation in the DUGL at the CJEM. The rock cover over the DUGL provides shielding equivalent to 4,000 m of water, which almost completely eliminates cosmic radiation^18^. Terrestrial radiation is emitted from natural radio nuclides present in varying amounts in the soil, air, water and other environmental materials. Radon, including ^222^Rn and ^220^Rn derived from terrestrial radioactive elements of uranium and thorium, is the most important component of natural radiation. Radon gas concentration was significantly higher but the γ radiation dose rate was significantly lower in the DUGL compared to the AGL. Other environmental parameters, including light, O_2_ levels, relative humidity, temperature, concentration of CO_2_ and air pressure can affect cell proliferation, but were unlikely to influence cell growth in the DUGL. Light, O_2_ levels, humidity, temperature, and concentration of CO_2_ were maintained at the same levels inside the CO_2_ incubators used for cell culture in the DUGL and the AGL. Air pressure could have affected biomass yield in cell cultures as cell growth rate is enhanced at 1.2–6 bar^19,20^. Air pressure in the DUGL was slightly higher than the AGL; however, the difference was reduced by the shift of gas and liquid as the cells were cultured in liquid. As much as possible to decrease batch effects, the analyses performed in both the DUGL and AGL also were on the same days.

Cells have evolved mechanisms for rapidly adjusting their biochemistry in response to changes in the environment, including radiation^21^. Most research has focused on the deleterious effects of acute, high or chronic radiation on cells, while some studies have demonstrated a stress response in cells grown at radiation doses that are 10 to 79 times lower than background^3^. In the present study, V79 cells cultured for 2 days in below-background radiation showed a changed protein profile. A total of 980 proteins were differentially expressed, including 576 proteins that were up-regulated and 404 proteins that were down-regulated, in cells cultured in the DUGL compared to the AGL. Over 70% of the DAPs identified by PRM were consistent with TMT proteomic analysis, implying that TMT proteomic analysis was reliable. These findings suggest protein synthesis was increased in V79 cells cultured in below-background radiation. Consistent with this, TEM of V79 cells cultured in the DUGL showed a hypertrophic ER and obvious Golgi bodies. GO analysis indicated that these DAPs exhibited a wide variety of cellular distributions and functions, which covered metabolic progress and macromolecular binding.

Ribosomal proteins play a critical role in ribosome assembly, protein translation, and cell proliferation. Some sources of extracellular stimulation (e.g. genotoxic chemicals, ionizing or ultraviolet radiation) can result in ribosomal stress and disturb ribosome biogenesis^22^. In the present study, GO enrichment analysis of DAPs showed that many terms (e.g. ribosome biogenesis, ribosome assembly) involved in ribosome biogenesis were significantly enriched. KEGG pathway analysis also suggested that the DAPs were involved in pathways of ribosome and ribosome biogenesis in eukaryotes. Among the terms enriched in GO and KEGG pathway analysis, ribosomal protein (RP) S3, RPS4, RPS14, RPS15, RPS27, RPL5, RPL6, RPL11, RPL23, RPL26, and RPL37 function to suppress cell proliferation by multiple mechanisms, including p-53ubiquitination and degradation, which leads to cell cycle and proliferation arrest^22^. In the PPI network, the core proteins were mainly ribosomal proteins, including RPS6, RPS14, RPS16, RPL8, RPL23, RPL3, and RPS18. These findings imply that ribosomal proteins played an essential role in the stress response in V79 cells caused by the deep underground environment and were involved in the multiple mechanisms that led to suppression of cell proliferation and cell survival under the changed environment^22^.

Spliceosome is a multi-megadalton ribonucleoprotein complex^23^. Splicing of precursor mRNA catalyzed by the spliceosome is an essential step in eukaryotic gene expression, by which noncoding sequences are removed and coding sequences are ligated together^23^. The spliceosome is central to the gene expression and protein synthesis required for cell growth and division^24^. In the present study, 28 DAPs were enriched in the splicesome pathway, and 158 DAPs were enriched in the ribonucleoprotein complex. Most of these proteins were upregulated in cells cultured in the DUGL. These data suggest that reduced background radiation altered gene expression by increasing spliceosome function, which helped V79 cells adapt to the changed environment.

Translation is an essential step in which genetic information is decoded to a functional polypeptide. Eukaryotic translation initiation factors (EIFs) are needed for the initiation phase of eukaryotic translation, helping to stabilize the formation of ribosomal pre-initiation complexes around the start codon, scan mRNA, and locate the initiation codon^25^. In the present study, 19 EIF protein subunits were up-regulated in V79 cells cultured in the DUGL, 14 of these proteins were involved in the RNA transport pathway and translation. Among those subunits, EIF2 attenuates the rate of translation in eukaryotic cells, allowing cells to conserve resources and initiate adaptive gene expression to restore cellular homeostasis^26^, and EIF3 can act as both a repressor and activator of translation. As stress proteins are controlled at the translational level^27^, upregulation of EFIs in response to low background radiation may allow selective translation of mRNAs to maintain the expression of stress proteins, while general protein synthesis is compromised.

Nucleic acid binding has a role in translation regulation. In the present study, GO enrichment analysis of DAPs showed nucleic binding proteins were significantly enriched. RNA-binding motif protein 3(RBM3) is a member of the glycine rich RNA-binding protein family that is induced by cold shock and low oxygen tension. RBM3 expression is essential for proper cell cycle progression and mitosis^28^. Cold-inducible RNA-binding protein (CIRP) helps cells to adapt to novel environmental conditions, such as UV radiation, by stabilizing specific mRNAs and facilitating their translation^29^. Both RBM3 and CIRP expression were increased in the cells cultured in the DUGL and indicated that these RNA-binding proteins might play some role in the stress of reduced background radiation.

The ER is a vital organelle with multiple functions, including protein synthesis and folding^17^. The ER can perceive and transduce environmental signals. ER stress activates the unfolded protein response (UPR), which leads to changes in key mediators of cell survival^30^. Recent research suggests that ionizing radiation can induce ER stress and initiate the UPR^31^. In the present study, 23/27 proteins enriched in the protein processing in ER pathway were down regulated in cells cultured in the DUGL. These included ER resident protein 29 (ERp29), protein disulfide isomerase A4 (PDIA4), endoplasmic reticulum chaperone BiP (BiP), also known as glucose-regulated protein 78 kDa (GRP78), and DNAJ homolog subfamily C member 3 (DNAJC3). ERp29 and PDIA4 are up-regulated in response to ER stress. GRP78 is an important molecular chaperone that prevents the aggregation of misfolded proteins in the ER^31,32^. DNAJC3 is a co-chaperone of GRP78 that attenuates general protein synthesis under ER stress^33^. This revealed that ER also involved in the stress of reduced background radiation. However, the most proteins down regulated in the cultures in DUGL need to be elucidated in future research.

Mitochondria play an essential role in cellular processes by producing ATP^34^ . Mitochondria are also involved in stress responses, and mitochondrial morphology reflects the energetic state and viability of cells^35^. Various environmental factors can affect mitochondrial morphology and metabolic activities (e.g. oxidative phosphorylation and programmed cell death), including laser or exogenous ROS-induced damage, which causes mitochondrial swelling^36^. In the present study, V79 cells cultured in the DUGL showed mitochondrial swelling, GO analysis revealed proteins enriched in the mitochondrial respiratory chain were dysregulated, and KEGG analysis of the DAPs showed the OXPPL pathway was significantly enriched. OXPPL is an important metabolic pathway that provides energy for cell growth and reproduction^37^. In V79 cells cultured in below-background radiation, 12/20 proteins enriched in the OXPPL pathway were down-regulated. This potentially altered energy homeostasis in V79 cells and their ability to proliferate. Consistent with these findings, Castillo et al. reported down-regulation of an ATPase in *S. oneidensis* cultured in low background radiation^21^.

Environmental stress induces the accumulation of reactive oxygen species (ROS) in cells as a host defense mechanism; however, ROS can cause oxidative stress if produced in excess^38^. In the present study, GO analysis showed enrichment of proteins involved in oxidoreductase activity and the oxidation–reduction process. This suggested that below-background radiation might induce oxidative stress. Consistent with this, Castillo et al.^6^ showed that *Shewanella oneidensis* cultured in low background radiation suffered oxidative stress, activated the SOS response (*katB* and *recA*) and up-regulated a putative metal efflux pump (*SOA0154*).

Our study has some limitations. First, we were unable to measure the levels of cosmic radiation in the DUGL at the CJEM and in the incubators. Second, one batch V79 cells were only maintained in the deep underground environment for a week and the experiments were conducted by a single research team. Longer term experiments investigating different phases of cell growth are required. Third, validation of differential expression of proteins in V79 cells cultured under low background radiation by knockdown and over expression studies should be conducted. Fourth, as ventilation in a deep mine is challenging, radiation was the only environmental factor that could be maintained at a constant level. Last, we expect that environmental factors other than below background radiation influenced V79 cell growth, but these remain to be elucidated.

In conclusion, proliferation of V79 cells was inhibited in the deep underground environment, likely because cells were exposed to reduced background radiation. There were apparent changes in the proteome profile of V79 cells cultured in the DUGL, which affected proteins related to the ribosome, RNA transport, translation, energy, metabolism, and gene spliceosome. These proteins may have induced cellular changes that delayed proliferation but enhanced survival, making cells adaptable to the changing environmental conditions. Our findings provide insight into the cellular stress response that is triggered in the absence of normal levels of radiation.

## Methods

### Environmental parameters in the DUGL and AGL

The DUGL at the CJEM is located in a goaf that is 820 m below sea level and under 1470 m of rock (Fig. 1). The AGL was constructed in an office in an administrative building near the entrance of the CJEM (altitude 590 m). Accessing the DUGL from the entrance of the CJEM requires a 1600 m walk and three elevators, which takes 1.5–2 h. Six environmental parameters [radon gas (1,027, Sunnuclear, USA), O_2_ (AR8100, Sigma, China), total γ ray dose rate (AT1121, Atomtex, Belarus) CO_2_, air pressure and relative humidity (Testo480,Testo, Germany)] in the DUGL and AGL were monitored at sites 1 m from the incubators, 0.5 m from the ground and 0.3 m from the palisades (DUGL)/wall(AGL). To minimize the effect of natural light on cell growth, the windows of the AGL were covered with black material and the room was illuminated with the same fluorescent lamps as the DUGL 24 h/day. All measurements and the following experiments were conducted under ventilated air.

### Cell culture

**Figure 8:**
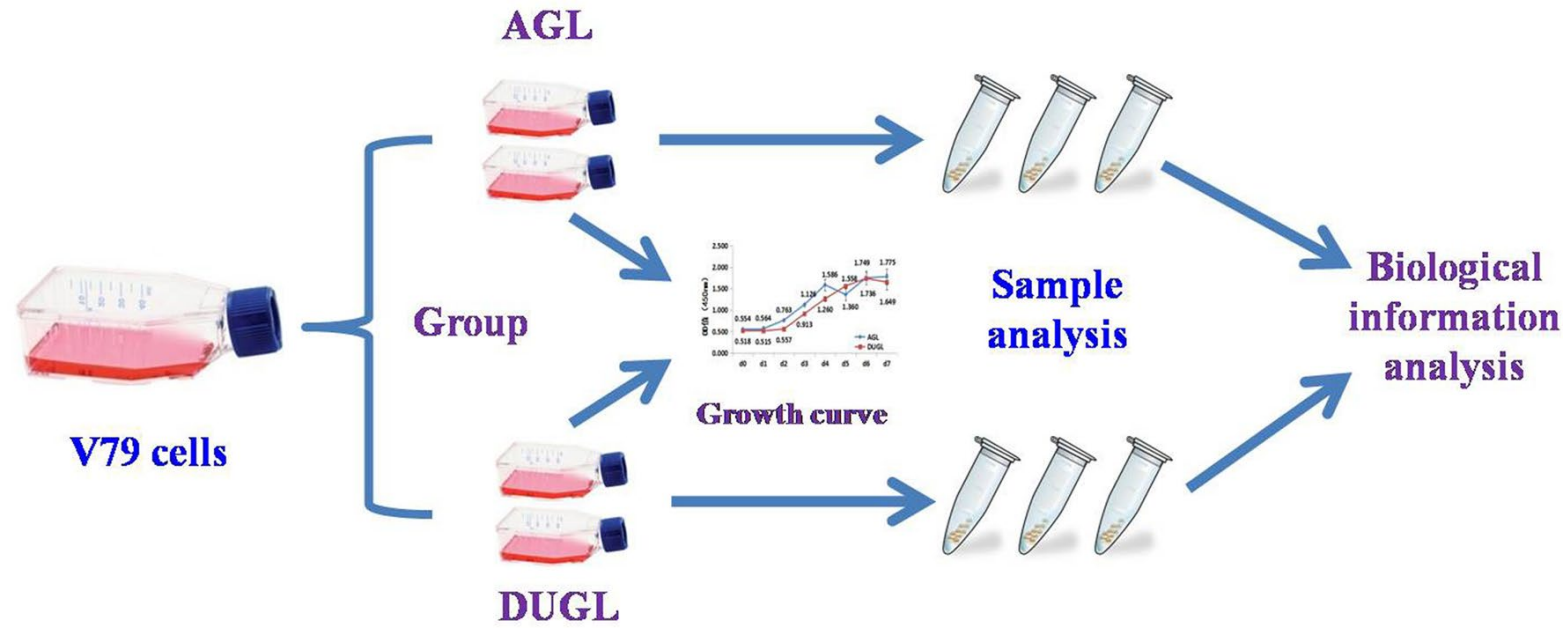
Flow chart showing the study design. *AGL* above-ground laboratory, *DUGL* deep underground laboratory.

Frozen Chinese hamster V79 lung fibroblast cells (Shanghai Enzyme-linked Biotechnology, China) were resuscitated and cultured in Dulbecco's modified eagle medium (DMEM) (Gibco, USA) supplemented with 10% foetal calf serum (Gemini, USA), 50 U dm^−3^ penicillin and streptomycin (Gibco, USA). When the cells were > 80% confluent, passaging was performed, and cultures were divided between four bottles, which were randomly assigned to be cultured in the DUGL or AGL. After one passage and two days of growth, three bottles from each location were frozen for use in proteomic analyses. The study design is summarized in Fig. 8. These experiments, which included cell proliferation, sampling of TEM and TMT, were conducted on the correspondingly same days in the DUGL and AGL to decrease batch effect.

### Cell proliferation

Cell proliferation in the cultures in the DUGL and AGL was measured by inoculating cell suspension into 96-well plates (5 × 10^5^ cells/ml, 200 µl/well). Plates were cultured at 37 °C in 5% CO_2_. 10 µL CCK-8 (MCE, USA) was added to the wells, and plates were incubated for 4 h at 37 °C in 5% CO_2_. Absorbance at 450 nm (OD_*450*_ nm) was measured. At each location, cell proliferation in five duplicate wells was measured daily over 7 days.

### Transmission electron microscopy

For TEM, V79 cells were cultured in the DUGL or AGL for two days. Cells were fixed with 2.5% glutaraldehyde at each location. Samples fixed in the DUGL were transported above ground for further analysis. Samples from both locations were washed five times for 20 min each at 4 °C with pre-chilled phosphate buffered saline (PBS). Cells were fixed with 1% OsO_4_ for 5 h at 4 °C. Cells were dehydrated in a graded series of ethanols(30%, 50%, 70%, 90%, 100%) and embedded in epoxy resin. Ultra-thin (80 nm) sections were cut and stained with 3% uranyl acetate and lead citrate. Sections were observed using a Hitachi H7650 TEM (Hitachi, Japan).

### Tandem mass tag protein quantification

Protein lysis buffer [7 M urea, 4% SDS, 30 mM HEPES,1 mM phenylmethanesulfonyl fluoride (PMSF), 2 mM EDTA, 10 mM dithiothreitol (DTT), 1 × protease inhibitor cocktail (Sigma-Aldrich, USA)] was added to samples of V79 cells cultured in the DUGL or AGL (n = 3 bottles in each location). Lysates were sonicated (Q800R, Qsonica, Newton, Connecticut, USA) on ice (5 s pulse on, 15 s pulse off, 180 W of power, 10 min) and centrifuged for 10 min at 10,000 × *g* and 4 °C. Protein concentrations in the supernatants were analyzed using a bicinchoninic acid (BCA) Protein Assay kit (Fisher Scientific, USA). 100 µg of lysed protein from each sample was reduced with DTT (final concentration, 10 mM), and alkylated with 35 mM iodoacetamide in the dark for 30 min. DTT was added to a final concentration of 10 mM and samples were incubated at room temperature for 10 min, to quench excess iodacetamide. Samples were incubated with pre-chilled (-20 °C) acetone for 3 h and centrifuged for 30 min at 20,000 × *g* and 4 °C. Samples were washed twice with a 50% acetone and 50% ethanol mix and centrifuged for 30 min at 20,000 × *g* and 4 °C. Precipitates were resuspended with 100 µl 100 mM TEAB and digested twice (for 4 h and 12 h) with trypsin (Promega, USA) at 37 °C using an enzyme-protein ratio of 1.0:100 (w/w).

TMT label reagents were equilibrated to room temperature, dissolved in 41 µl of anhydrous acetonitrile, and centrifuged. Digested samples were labeled with the 6 plex TMT tag (Thermo Scientific, USA) for 2 h at room temperature. Samples from the DUGL were labeled with TMT-126, TMT-127, and TMT-128. Samples from the AGL were labeled with TMT-129, TME-130, and TME-131. After labeling, samples were combined, lyophilized to dryness, and desalted on a Sep-PakC18 column (100 mg, 1 cc, Waters, USA)^39^ .

Subsequently, labeled samples were fractionated by high performance liquid chromatography (HPLC) using a BEHC 18 column (2.1 × 150 mm, 1.7 μm, 130 Å) (Acquity UPLCBEH C18, Waters Corporation, Eschborn, Germany) and a two-mobile-phase gradient elution system (mobile phase A: 10 mM ammoniumformate, pH 10; mobile phase B: 10 mM ammoniumformate and 90% acetonitrile, pH 10). The elution gradient was: 0–5 min 5% B, 5–95 min 5–30% B, 95–105 min 30–80% B, 105–105.1 min 5% B, 105.1–120 min 5%-stop. The flow rate was 300 nL/min. The absorbance wave length was set to 215 nm. Eluted fractions were collected by an automated fraction collector and combined into 12 fractions.

Peptides were analyzed by liquid chromatography-tandem mass spectrometry (LC–MS/MS) on an Orbitrap Fusion mass spectrometer (Thermo Scientific, San Jose, CA, USA) using higher-energy C-trap dissociation (HCD), positive ionization mode and a data dependent acquisition (DDA) strategy, which involved automatically switching between full spectrum MS mode and full-spectrum product-ion (MS–MS) analysis mode. Settings for full spectrum MS mode were: ESI voltage, 2 kV; capillary temperature, 300 oC; automatic gain control (AGC) target, 5 × 10^5^; resolution, 70,000; scan range, 350-1600 m/z; and maximum injection time, 50 ms. MS/MS acquisition targeted the 15 most intense parent ions. The settings were: resolution, 17,500 at m/z 200; MS/MS minimum ionic strength, 50,000; maximum injection time, 150 ms; AGC target, 2 × 10^5^, and isolation window, 2 Da. Ions with charge states 2 + , 3 + , and 4 + were sequentially fragmented by HCD with a normalized collision energy (NCE) of 30%. In all cases, one scan was recorded using dynamic exclusion of 30 s.

### Protein identification and quantification

Raw data were processed using Proteome Discoverer (PD) (Version 1.4.0.288, Thermo Fisher Scientific, USA), and proteins were identified using MASCOT (Version 2.3.2, Matrix Science). MASCOT was set up to search the Uniprot database (Taxonomy: CricetulusGriseus, 34,954 entries) assuming the digestion enzyme trypsin. MASCOT was searched with a fragment ion mass tolerance of 0.050 Da and a parent ion tolerance of 10.0 PPM. Carbamidomethyl of cysteine, TMT 6plex on lysine residues, and the n-terminus were specified in MASCOT as fixed modifications. Oxidation of methionine was specified in MASCOT as a variable modification. Relative quantification of identified proteins was determined according to the weighted ratios of the uniquely identified peptides that belonged to a specific protein. Parameters for protein identification and quantification were as previously reported^40^, except the false discovery rate (FDR) was ≤ 1%. A paired *t* test was performed to determine statistical significance between the DUGL and AGL. Proteins with a *p* value < 0.05 and an absolute fold change ≥ 1.2 were considered differentially expressed.

### Parallel reaction monitoring

PRM performed on a Triple TOF 6600 + LC–MS/MS system was used to verify TMT results. Proteins were extracted, lysed and desalted as previously described. DDA raw files were analyzed with MaxQuant (version 1.3.0.5) using the default settings. Resulting data were searched against the UniProt-cricetulus + griseus.fasta database using Protein Pilot. PRM validation data were analyzed using Skyline; peak shapes for target peptides were manually inspected.

### Biological function

GO annotations and KEGG classifications were performed by a multi-omics data analysis tool, OmicsBean software (https://www.omicsbean.com:88/). GO terms and KEGG pathways statistics were performed by Fisher’s exact test with a corrected *p* value < 0.05 considered as significantly enriched. To further ascertain functional interactions between DAPs, PPI networks were constructed using Cytoscape software**.** With a confidence cutoff of 400; interactions with larger confidence scores are indicated with solid lines between proteins.

### Statistical analysis

A normality test was used to determine if environmental data were normally distributed. Normally distributed data are expressed as mean ± standard deviation (SD). Non-normally distributed data are expressed as median (interquartile range). Differences in environmental characteristics between the DUGL and AGL were compared with the Students’*t*-test for normally distributed data and the rank sum test for non-normally distributed data. A *p* value < 0.05 was considered statistically significant.

## Supplementary information

Supplementary Tables.

Supplementary Legends

## Data Availability

All data in this study are included in the article and its supplementary files. The mass spectrometry proteomics data have been deposited to the ProteomeXchange Consortium (Subproject: SRR11515332-7).
