# Supplementary material for: Proteomics provides insights into the inhibition of Chinese hamster V79 cell proliferation in the deep underground environment
Source: Sci Rep. 2020 Sep 10;10:14921. doi: 10.1038/s41598-020-71154-z (PMC7483447; doi:10.1038/s41598-020-71154-z)
Supplement: Supplementary file 2 — Supplementary Legends [file 41598_2020_71154_MOESM2_ESM.docx]

**Supplementary TableS** 1 Differentially abundant proteins.

**Supplementary TableS 2** Gene ontology analyses of differentially abundant proteins(Top 50).

**Supplementary TableS** 3. Differentially abundant proteins verified by Parallel Reaction Monitoring.
